# Effectiveness of intraoral splints in the treatment of migraine and tension‐type headache: A systematic review

**DOI:** 10.1002/cre2.779

**Published:** 2023-09-07

**Authors:** Olga Martínez‐Álvarez, Eduardo Wojtovicz, Jose Luís De la Hoz, Juan Mesa, Susan Armijo‐Olivo

**Affiliations:** ^1^ San Pablo Ceu University Madrid Spain; ^2^ Faculty of Business and Social Sciences University of Applied Sciences Osnabrück Germany; ^3^ Department of Physical Therapy, Faculty of Rehabilitation Medicine University of Alberta Edmonton Alberta Canada

**Keywords:** headache disorders, migraine disorders, splints, tension‐type headache

## Abstract

**Objectives:**

The main objective of this systematic review was to assess the effectiveness of intraoral splints in treating migraine and tension‐type headaches.

**Material and Methods:**

The article search was conducted within seven electronic databases (Medline, PubMed, Embase, CINAHL PLUS with full text, Cochrane Library Trials, Web of Science, and Scopus) with no date limits or language restrictions up to June 12, 2022. Strict inclusion and exclusion criteria were set for article selection. At the same time as data extraction, each study's risk of bias (RoB) was evaluated using the Cochrane tool to assess their RoB. Subsequently, the Cochrane Grading of Recommendations Assessment Development and Evaluation was used to evaluate the certainty of the evidence.

**Results:**

Four controlled clinical trials were included. These trials were heterogeneous in terms of (1) diagnosis, (2) design of the intraoral splints, and (3) tools for reporting the results, which made it difficult to compile the data as well as evaluate its quality. Trials reported a reduction in the frequency of headache and pain intensity when using intraoral splints; however, this therapy was not superior to medications.

**Conclusions:**

The evidence is very low for the use of oral splints as a therapeutic alternative to medication in the treatment of migraine and/or tension‐type headache.

## 1 INTRODUCTION

Migraine and tension‐type headaches are the most prevalent neurological disorders (Troeltzsch et al., 2014) and one of the sixth most disabling diseases worldwide (Coppola et al., 2016). According to the International Classification of Headache Disorders‐3rd edition (ICHD‐3), a headache could be a symptom of multiple pathologies (secondary headache), or be a disease itself (primary headache; IHS, 2018). Migraine appears to arise from the activation and sensitization of trigeminal nociceptors in the meninges and their respective blood vessels. Calcitonin gene‐related peptide (CGRP) could be involved in the pathogenesis of migraine and cluster headaches. The mechanisms that lead to peripheral and central sensitization are relevant in the chronification of both migraine and tension‐type headaches. The hypothalamus, the upper center of the nervous system, could be important in the onset of migraine attacks (Gaul et al., 2017).

Headaches commonly coexist with temporomandibular disorders (TMDs), and therefore their diagnosis becomes complicated by overlapping symptoms between these two conditions (Tfelt‐Hansen et al., 1981). For example, electromyographic studies have shown an increase in the electrical activity of the pericranial muscles in patients with acute migraine attacks compared with healthy subjects, which has also been seen in patients with TMDs (Bakal & Kaganov, 1977; Tfelt‐Hansen et al., 1981).

In addition, the treatment of headaches and TMDs share similar options due to comorbidity between both entities and similar pain distribution patterns (Lim et al., 2010). Until now, the prevention and treatment of both clinical entities include using medication with complementary or alternative therapies such as nutrition, physical therapy, neuromodulation, or intraoral splints (Fernández‐de‐Las‐Peñas et al., 2020; Troeltzsch et al., 2014). Intraoral splints have been suggested as a nonpharmacological alternative to manage headaches based on the positive response of these devices in treating TMDs (Goncalves et al., 2013; Saha et al., 2019). The use of intraoral splints can have a beneficial effect on headaches, reducing their frequency and severity in combination or not with pharmacological treatment (Sletten et al., 2015; Troeltzsch et al., 2014). However, studies that investigated their effectiveness have yielded contradictory results, and it is unclear their role in these conditions (Costa et al., 2015; Goncalves et al., 2013). Different designs of intraoral appliances have been used, from partial anterior splints (Bruno & Krymchantowski, 2018) to full arch splints (Goncalves et al., 2013). Theories about the mechanism of action of intraoral splints have been thought that a reduction in the stimulus originating in the masticatory musculature can reduce central sensitization, reducing the headache frequency (Wright & Jundt, 2006).

Only two systematic reviews on the use of intraoral splints have been published (Manrriquez et al., 2021; Stapelmann & Türp, 2008). The review carried out by Stapelmann and Türp analyzed the use of the Nociceptive Trigeminal Inhibition Tension Suppression System (NTI‐tss) in the joint management of bruxism, TMD, and primary headaches (Stapelmann & Türp, 2008). Therefore, to our knowledge, no previous review focused on the effectiveness of the NTI‐tss oral appliances for primary headaches in isolation. Regarding the second review (Manrriquez et al., 2021), they analyzed the reduction of headache intensity and frequency with stabilization splints in patients with TMD and headache comorbidity. This work included primary and secondary headaches to TMDs and craniomandibular disorders without distinction; thus, the headache diagnosis was not clearly identified (Manrriquez et al., 2021). A specific diagnosis must be performed in patients who suffer headaches based on recognized diagnostic criteria or confirmed by a neurologist. The pathophysiological mechanism differs significantly between primary headaches and those associated with TMD (Ashina et al., 2005; Do et al., 2018; Iyengar et al., 2017), so they should not be evaluated together. A more specific research question focusing only on these primary conditions would be important to clarify the existing controversy regarding its effectiveness to manage them. It is crucial to verify the efficacy of noninvasive treatments, such as using intraoral appliances for migraine and tension‐type headaches, since these are clinical entities with high annual healthcare costs and whose drug therapy can cause over‐medication and unwanted side effects (Franco et al., 2011).

Therefore, the aims of this systematic review were to summarize and analyze the evidence from randomized controlled trials (RCTs) and clinical trials that investigate the effectiveness of intraoral splints in reducing the frequency and intensity of symptoms in migraine and tension‐type headaches and to evaluate their methodological quality and risk of bias (RoB).

## 2 MATERIALS AND METHODS

This project was registered in PROSPERO (CRD42021260195) and reported based on the PRISMA guidelines (Page et al., 2021).

### 2.1 Search strategy

The present systematic review was limited to parallel randomized clinical trials and controlled trials. Searches were carried out in the following databases: Medline (Ovid MEDLINE), PubMed, Embase (Ovid interface), CINAHL PLUS with full text (EBSCOhost interface), Cochrane Library Trials (Wiley interface), Web of Science (Indexes = SCI‐EXPANDED, SSCI, A & HCI, ESCI), and Scopus (Elsevier). Searches were last updated on June 12, 2022. No date limits were applied to the databases or restrictions on language. MeSH terms were consulted in the health sciences descriptors (DeCS) and free terms. A manual search was conducted by reviewing the list of references and items cited from the included articles using Web of Sciences database. The search strategies are described in Supporting Information: Appendix 1.

### 2.2 PICOS principle and selection criteria

This systematic review was conducted to answer the following clinical question: “Are intraoral splints effective in reducing the intensity, frequency, and disability of migraine and tension‐type headache compared to other pharmacological and nonpharmacological therapeutic alternatives?” The population, intervention describing the two splint designs included in this review (Moufti et al., 2007), control and outcomes (PICO) question, and eligibility criteria are detailed in Table 1.

**Table 1:** PICO question and eligibility criteria.

Inclusion criteria		Exclusion criteria	
Population	>18 years. Diagnosis of migraine or TTH with ICHD‐3 or previous versions. Neurologist With or without comorbidities.	Population	Teenagers and children. Taking medications that affect the CNS, alcohol, and drug abuse, and painful odontogenic disorders. There is no exclusion for any type of diagnosis or associated comorbidity.
Intervention	Two specific designs of intraoral appliances: 1. *Stabilization splint*: A removable, full coverage, and transparent resin device with a flat surface (unmarked tooth prints on its surface), made to measure and placed on the upper or lower teeth. This design presents the ideal occlusion characteristics: − Multiple even contacts on posterior teeth in the retruded contact position with lighter contacts on anterior teeth. − Anterior guidance ramp providing canine guidance and disocclusion of the posterior teeth in lateral and protrusive excursions. 2. *NTI‐tss*: A removable, partial coverage and resin device limited to the incisors region with a flat surface, made to measure and placed on the upper teeth. It is only registered for lateral and protrusive excursions (Moufti et al., 2007).	Intervention	Soft, thermoplastic material, deformable splint, and any splint design that does not meet the characteristics described in the inclusion criteria.
Comparation	No intervention, placebo, sham intervention, or any type of treatment.	Comparation	‐
Outcomes	Changes in headache frequency Changes in headache intensity Changes in headache disability	Outcomes	‐
Studies	Parallel randomized clinical trials and controlled trials. All languages. Publication date not limited.	Studies	Cases and controls, cohorts, descriptive studies, qualitative research, systematic reviews, meta‐analyzes, narrative reviews, case series, reports, protocols, pilot studies, comments, or letters to the editor

Abbreviations: CNS, central nervous system; ICHD‐3, International Classification of Headache Disorders‐3rd edition; NTI‐tss, Nociceptive Trigeminal Inhibition Tension Suppression System; TTH, tension‐type headache.

### 2.3 Selection of the studies

After the search, the records were imported into Endnote reference manager and subsequently to Covidence web platform. A manual check was made of the duplicates removed during these processes to verify accuracy. Covidence web platform was used to establish contact between reviewers during study selection and data extraction. At the same time, it allowed to eliminate duplicates, maintain blinding, and facilitate the process.

Two reviewers (O.M.A. and E.L.W.) independently selected the abstracts, titles, and full texts, according to the eligibility criteria (Table 1). Any disagreement between the two main reviewers required a re‐evaluation of those articles to reach an agreement to include or not the study. Once the articles were selected for extraction, a manual search and a citation track were carried out using Web of Sciences database. That is, the references of the selected articles were reviewed, as well as who cited them for locating possible articles that would meet the criteria for inclusion.

### 2.4 Data extraction

Data extraction was performed in a pilot‐tested Excel form developed especially for this review. Drop‐down menus were used whenever possible to standardize the extracted data.

An independent reviewer (O.M.A.) extracted and organized the data in Excel for further analysis and synthesis. A second reviewer (E.L.W.) verified all the extracted data. In case of discrepancies between reviewers, a consensus meeting was conducted. If there was no consensus, a third reviewer made the decision (S.A‐O). Articles were analyzed with the information published. Data extracted included but was not limited to *article information* (e.g., author name, year of publication, and language); *population information* (e.g., age, sex, ethnicity, and diagnosis); *study information* (e.g., primary objective, study design, sampling method used, sample size calculation, and diagnostic tool); *treatment characteristics* (such as type of intervention, description of the intervention, whether the intervention was applied alone or in combination, and duration of the entire treatment); *results* (e.g., estimates and analysis); *conclusion*; *limitations/comments*; and *recommendations*.

### 2.5 Assessment of the RoB of the studies

The quality of the studies was assessed with the RoB‐2 tool for RCTs (Cochrane Methods Bias). Two reviewers independently (O.M.A. and E.L.W.) evaluated each domain for each study and supported their claims, using the most detailed information possible. An agreement had to be reached between the two reviewers. The studies were evaluated with the following domains: (1) random process, (2) deviations from planned interventions, (3) missing outcome data, (4) outcome measurement, and (5) selection of the reported outcome. For the overall RoB assessment, the following decision rules were established:

− **Low RoB**: The study is judged to be at low RoB for all domains of the RoB tool.
− **Some concerns**: The study is judged to raise some concerns in at least one domain of the RoB tool but not at high RoB for any domain.
− **High RoB**: The study is judged to be at high RoB in at least one domain of the RoB tool or to have some concerns for multiple domains in a way that substantially lowers confidence in the results.

### 2.6 Data synthesis

Data were synthesized qualitatively using evidence tables and figures. Forest plots were performed to display estimates of individual studies and the direction of effects. The information was organized based on diagnosis (i.e., migraine and TTH) and outcomes of interest. Due to the high heterogeneity of included studies, and the limited number of them, a meta‐analysis was not feasible (as planned in our protocol). The overall quality of the evidence and certainty of this review was evaluated with the GRADE tool. The evidence was classified as high, moderate, low, or very low (Guyatt et al., 2008). The domains that can decrease the quality of the evidence are (1) limitations in the design, (2) inconsistency between the results of different studies, (3) indirectness, (4) imprecision of effect estimators, and (5) publication bias.

## 3 RESULTS

### 3.1 Selection of studies

**Figure 1:**
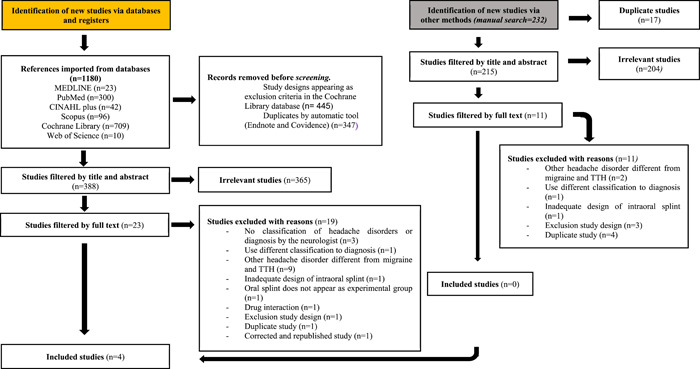
PRISMA flow diagram. Flowchart reporting the number of articles identified in each database and subsequent manual search, with the different selection phases and reasons for excluding those not admitted. TTH, tension‐type headache.

The PRISMA flow chart reflects the process that has been carried out in the selection of the studies (Figure 1). After the first screening of titles and abstracts, 23 articles were selected for their full‐text screening. Nineteen studies did not meet the inclusion criteria established for which they were excluded, clearly specifying the reasons for the exclusion (Supporting Information: Appendix 2). Subsequently, only four studies were selected for inclusion in this systematic review (Bruno & Krymchantowski, 2018; Ekberg & Nilner, 2006; Goncalves et al., 2013; Shankland, 2002). Finally, 232 articles were identified through the manual search; however, none of them met the inclusion criteria, and thus no new article was added from the manual search.

### 3.2 Study characteristics—Synthesis of results

#### 3.2.1 General characteristics of included studies

Four studies (Bruno & Krymchantowski, 2018; Ekberg & Nilner, 2006; Goncalves et al., 2013; Shankland, 2002) described as randomized control trials were included; two included patients with migraine (Bruno & Krymchantowski, 2018; Goncalves et al., 2013), one included patients with tension‐type headache (Ekberg & Nilner, 2006), and another one included patients with both clinical entities (Shankland, 2002). The studies were carried out in universities (Ekberg & Nilner, 2006), hospitals (Bruno & Krymchantowski, 2018), or private clinics (Goncalves et al., 2013), except for one that did not report its setting (Shankland, 2002). Both more recent studies (Bruno & Krymchantowski, 2018; Goncalves et al., 2013) used the ICHD‐2 (Olesen, 2004) as diagnostic criteria; and the other two studies (Ekberg & Nilner, 2006; Shankland, 2002) used the IHS (Ravishankar, 2010). No study used the criteria of the ICHD‐3 (IHS, 2018). Table 2 shows the main characteristics of the studies included in the present review.

**Table 2:** Summary of interventions, demographics, and adverse events.

Reference	Bruno and Krymchantowski (2018)	Goncalves et al. (2013)	Ekberg and Nilner (2006)	Shankland (2002)
Sex	10♂/66♀ (final sample)	89♀ (final sample)	8♂/52♀ (initial sample)	Mixed (does not distinguish gender)
Age	39.45 (11.11)	34.3 (8.8)	29 (Costa et al., 2015)	No information
Diagnosis	Migraine	Migraine	Tension‐type headache	Migraine and tension‐type headache
Diagnosis tool	ICHD‐2	ICHD‐2	IHS	IHS
Comorbidities	Not existing	Mixed: TMD	Mixed: TMD	Mixed: Bruxism
Number of groups	3	4	2	2
Sample	Initial: 90/Final: 76	Initial: 94/Final: 89	Initial: 60/Final: 40	Initial: 94/Final: 94 (no dropouts are reported)
Therapeutic comparison; *n*	− Amitriptyline; *n* = 28 − NTI‐tss (intraoral splint): *n* = 25 − Nonocclusal splint* (acrylic resin palatal coverage device made in such a way that it does not interfere with the normal occlusion of the patient ‐ sham device); *n* = 23	− Stabilization splint (Michigan); *n* = 23 − Stabilization splint (Michigan) + propranolol; n = 22 − Propranolol + nonocclusal splint* (sham device); *n* = 23 − Placebo drug + nonocclusal splint* (sham device); *n* = 21	− Stabilization splint (Michigan); *n* = 30 − Nonocclusal splint* (sham device); *n* = 10	− NTI‐tss (intraoral splint): *n* = 51 − Control splint** (custom acrylic retainers in the shape of the patient's teeth made of 0.02‐inch acrylic‐sham device); *n* = 43
Study period	3 months (single evaluation at the end of the study)	3 months (single evaluation at the end of the study)	10 weeks/6 months/12 months (end of the study)	8 weeks (single evaluation at the end of the study)
Variables (tool; unit)	Frequency (daily calendar; episodes/week)	Frequency (daily calendar; days/month) Intensity (VAS; mm) Disability (MIDAS; score)	Frequency (verbal questionnaire; description)	Intensity (VAS, mm) Frequency (daily calendar; percentage of daily time)
Adverse events	None adverse events	None reported	None reported	None reported
Results	Amitriptyline was superior to NTI‐tss and non‐occlusal splints. The NTI‐tss was not superior to a sham splints.	No differences between stabilization splints and sham in terms of frequency, intensity, and disability.	A significant reduction of headache frequency was obtained in patients receiving stabilization splints at 10 weeks when compared to the sham non‐occlusal splint.	The NTI‐tss group presented a statistically significant reduction for migraine and TTH when compared with the control splints.

Abbreviations: HIS, International Headache Society; ICHD‐2, International Classification of Headache Disorders‐2nd edition; MIDAS, migraine disability assessment; NTI‐tss, Nociceptive Trigeminal Inhibition Tension Suppression System; TMD, temporomandibular disorders; TTH, tension‐type headache; VAS, Visual Analogue Scale.

The results regarding the effectiveness of intraoral splints versus the control group and other interventions by each outcome of interest are provided below.

### 3.3 Migraine

#### 3.3.1 Headache outcomes (frequency, intensity, and disability): Intraoral splints versus sham splints at the end of treatment

**Figure 2:**
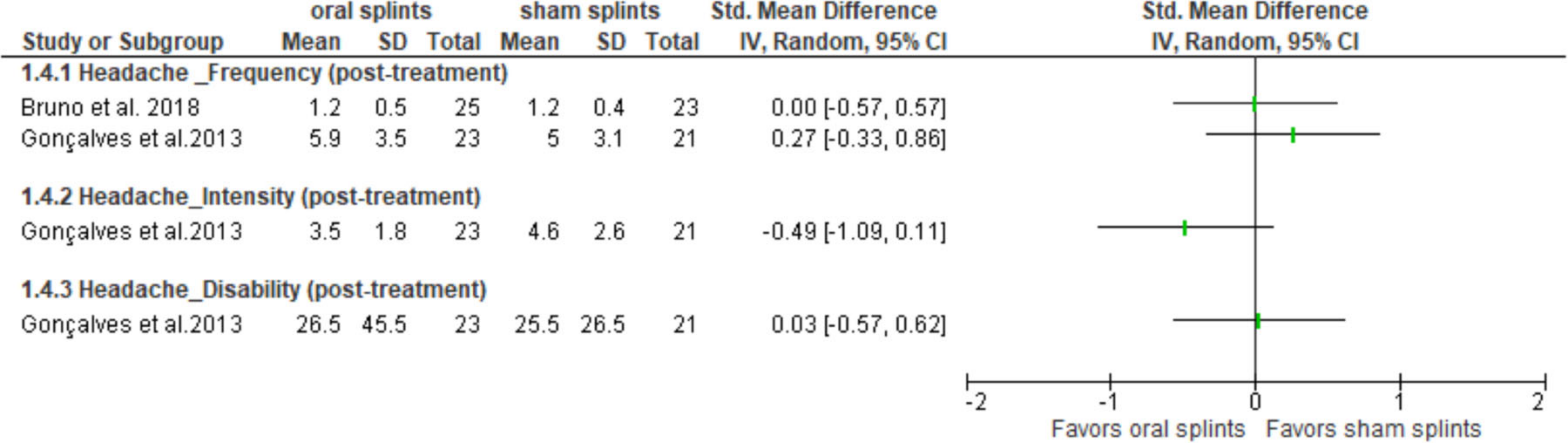
Descriptive forest plot showing posttreatment changes between oral splints versus sham splints after 3 months in patients with migraine. CI, confidence interval; SD, standard deviation; Std, standard.

Three studies (Bruno & Krymchantowski, 2018; Goncalves et al., 2013; Shankland, 2002) looked at the effectiveness of intraoral splints versus sham splints in patients with migraine, but only two provided quantitative data (Bruno & Krymchantowski, 2018; Goncalves et al., 2013) (Figure 2). Of these studies, all investigated frequency of migraine, two authors (Goncalves et al., 2013; Shankland, 2002) looked at intensity, and only Gonçalves et al. (2013) investigated disability related to migraine (Table 3).

**Table 3:** Summary of the direction of effect of the included studies at the different study times (using quantitative and qualitative data).

Author, year	Bruno and Krymchantowski (2018)	Goncalves et al. (2013)	Ekberg and Nilner (2006)	Shankland (2002)
Oral splint versus Sham splint				
*Migraine*				
Frequency of headache (8 weeks)	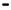	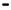	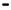	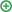
Intensity of headache (8 weeks)	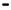	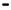	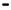	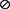
Disability of headache (8 weeks)	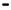	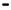	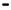	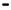
Frequency of headache (3 months‐at the end of treatment)	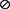	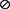	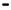	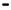
Intensity of headache (3 months‐at the end of treatment)	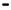	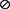	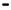	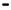
Disability of headache (3 months‐at the end of treatment)	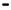	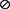	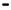	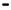
*Tension‐type headache*				
Frequency of headache (8–10 weeks)	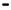	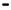	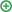	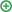
Intensity of headache (8–10 weeks)	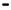	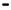	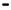	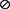
Disability of headache (8–10 weeks)	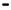	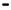	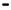	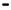
Frequency of headache (6 months)	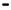	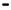	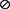	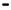
Intensity of headache (6 months)	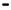	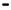	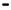	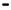
Disability of headache (6 months)	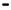	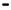	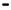	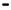
Frequency of headache (12 months‐at the end of treatment)	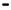	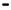	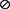	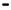
Intensity of headache (12 months‐at the end of treatment)	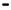	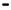	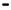	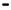
Disability of headache (12 months‐at the end of treatment)	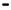	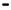	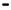	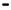
Oral splint versus others treatments				
*Migraine*				
Frequency of headache (3 months‐at the end of treatment)	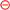	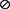	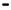	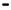
Intensity of headache (3 months‐at the end of treatment)	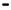	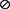	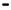	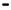
Disability of headache (3 months‐at the end of treatment)	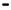	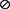	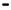	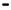
*Tension‐type headache*				
Frequency of headache	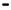	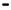	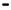	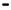
Intensity of headache	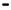	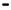	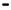	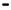
Disability of headache	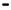	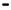	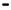	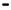

*Note*: 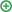 Favoring the orthosis oral of interest; 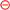 Favoring the comparison; 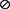 No difference between comparisons; 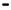 Outcome not evaluated

When analysing the *frequency of migraine*, two of the studies provided quantitative data, and their results are displayed in Figure 2 (Bruno & Krymchantowski, 2018; Goncalves et al., 2013). None of them could find a statistically significant difference between intraoral splints and sham splints, although Gonçalves et al. (2013) showed a slight tendency to favor the sham splints. Although Shankland et al.'s (2002) study did not provide quantitative data to look at the estimates, the authors reported a statistically significant difference in migraine frequency in favor of the intraoral splints (Table 3).

Two studies (Goncalves et al., 2013; Shankland, 2002) analyzed the *intensity of migraine* after 8 weeks (Shankland, 2002) and 3 months of treatment (Goncalves et al., 2013). Only Gonçalves et al. (2013) provided quantitative data; their results are displayed in Figure 2. Both studies did not find any statistical or clinically significant difference between patients receiving intraoral splints or sham splints on the *intensity of migraine*; however, in both cases, there was a trend in favor of intraoral splints. No differences were observed for disability either, which was studied only by one study at 3 months (Goncalves et al., 2013) (Figure 2, Table 3).

#### 3.3.2 Headache outcomes (frequency, intensity, and disability): Intraoral splints versus other treatments at the end of treatment

**Figure 3:**
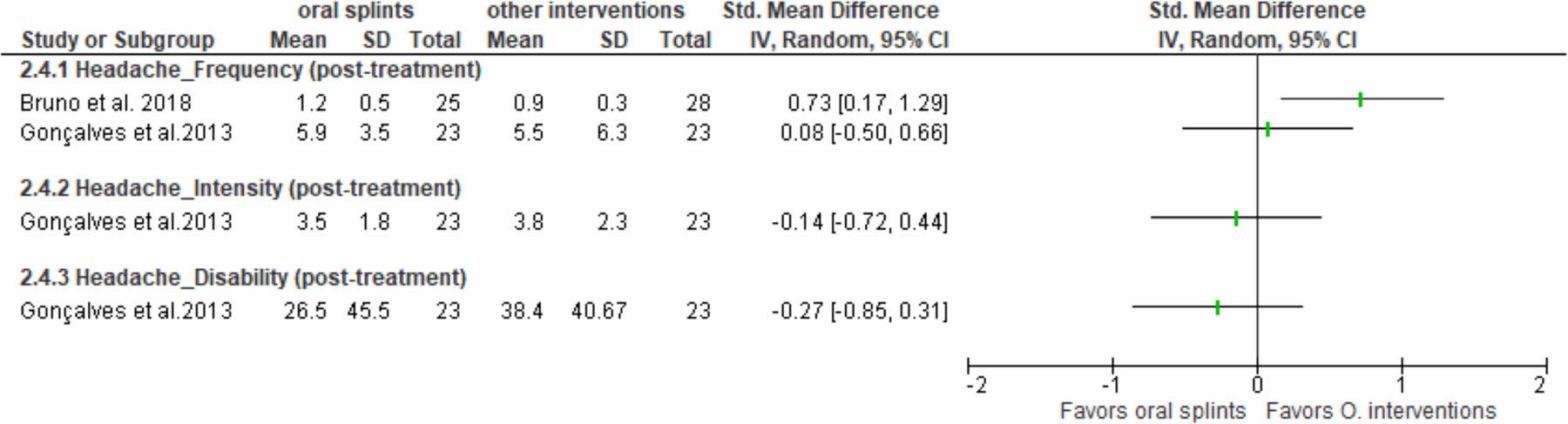
Descriptive forest plot showing posttreatment changes between oral splints versus other interventions after 3 months in patients with migraine. CI, confidence interval; SD, standard deviation; Std, standard.

Two studies (Bruno & Krymchantowski, 2018; Goncalves et al., 2013) looked at the effectiveness of intraoral splints versus other treatments in patients with migraine. In both studies, the comparison was made with pharmacological therapy. In the study by Bruno and Krymchantowski (2018), the group treated with amitriptyline presented a statistically significant reduction in the frequency of migraine, while in the study by Gonçalves et al., no statistically significant differences were observed between these treatments; however, there was a trend in favor of the pharmacological treatment (Goncalves et al., 2013) (Figure 3, Table 2).

Regarding intensity and disability, only one study looked at these outcomes (Goncalves et al., 2013). No statistically significant differences between splints and other interventions were found on these variables (Goncalves et al., 2013) (Figure 3, Table 3).

### 3.4 Tension‐type headache

#### 3.4.1 Headache outcomes (frequency, intensity, and disability): Intraoral splints versus sham splints at different times of the study

Two studies (Ekberg & Nilner, 2006; Shankland, 2002) examined the effectiveness of intraoral splints versus sham splints in patients with tension‐type headaches. Both studies analyzed the TTH frequency; only one evaluated intensity (Shankland, 2002), while neither evaluated disability.

Concerning the frequency, both studies found statistically significant differences between intraoral splints and sham splints in the short term; however, Ekberg and Nilner's (2006) study evaluated a longer follow‐up time, not maintaining statistical significance at 6 and 12 months of analysis.

Neither of the two articles provided quantitative data (i.e., mean or standard deviation (SD)), so a quantitative analysis was not possible. Based on these studies' results, intraoral splints reduced the frequency of TTH episodes in the short term. However, these results were not maintained at a long‐term evaluation. The dropout rate in the sham group (nonocclusal splints) was excessively high (66%) compared with the no dropouts in the treatment group; therefore, the reported results could be biased (Ekberg & Nilner, 2006).

The intensity of TTH was only evaluated by Shankland at 8 weeks of treatment, observing a more significant reduction in the intraoral splints group compared to the control group, but without being statistically significant (Shankland, 2002) (Table 3).

#### 3.4.2 Headache outcomes (frequency, intensity, and disability): Intraoral splints versus other treatments at different times of the study

In these two studies (Ekberg & Nilner, 2006; Shankland, 2002) that included patients with tension‐type headaches, the comparison group was a non‐occlusal splint, so no alternative treatments were analyzed (Table 3).

### 3.5 RoB

**Figure 4:**
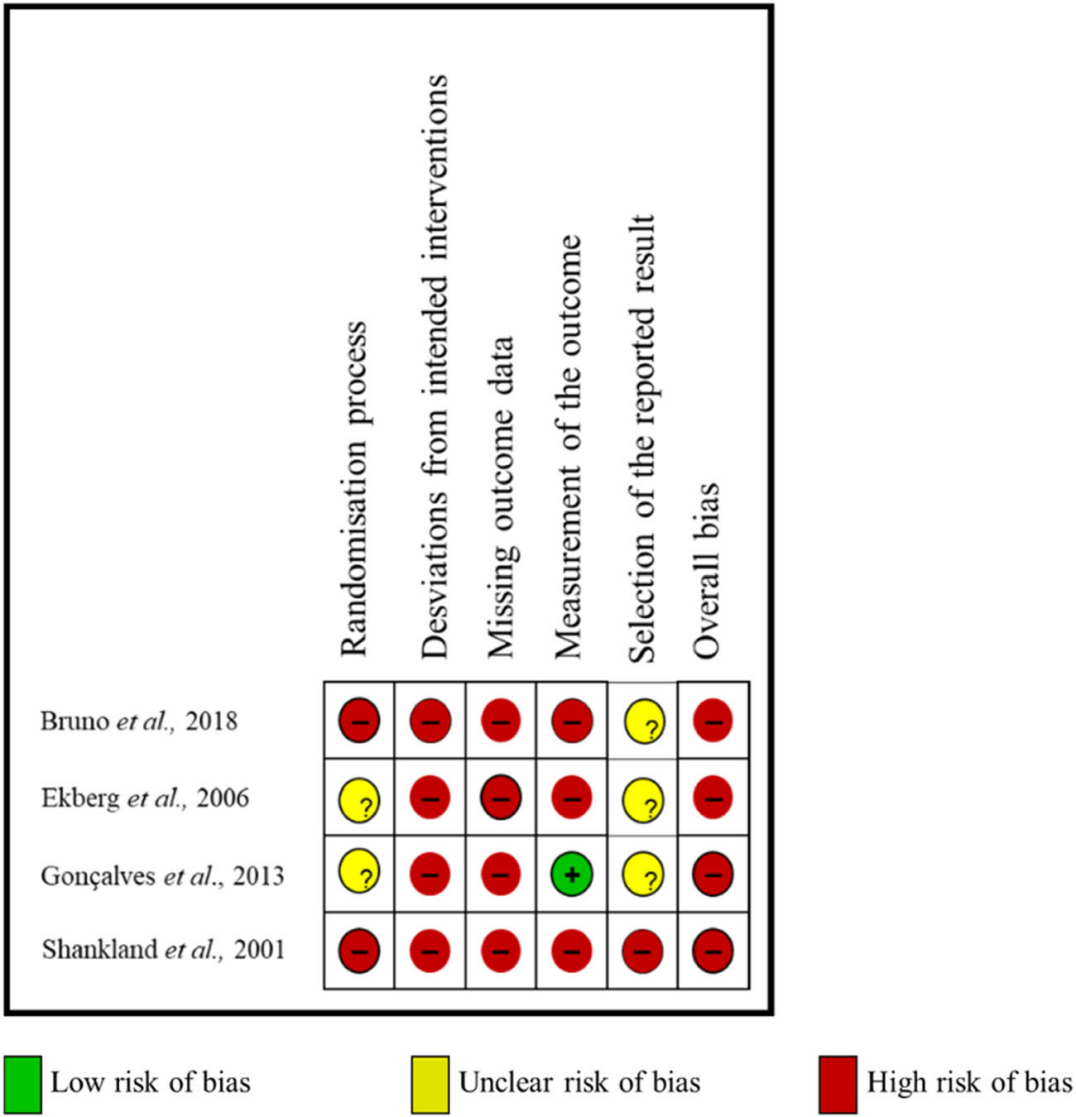
Risk of bias (RoB) of included studies using the RoB‐2 tool. Reviewers' judgments about each item of risk of bias for each included study.

The four studies included (Bruno & Krymchantowski, 2018; Ekberg & Nilner, 2006; Goncalves et al., 2013; Shankland, 2002) were considered to have a high RoB (Figure 4). The main concerns regarding these studies were a questionable randomization process needed to describe the process or directly provide information about it correctly. Three studies (Bruno & Krymchantowski, 2018; Ekberg & Nilner, 2006; Shankland, 2002) did not report clear blinding for all study groups, only the study by Gonçalves was considered a single‐blind study (Goncalves et al., 2013). Data loss could have affected the effect of the intervention in all four studies (Bruno & Krymchantowski, 2018; Ekberg & Nilner, 2006; Goncalves et al., 2013; Shankland, 2002).

### 3.6 Assessment of the quality/certainty of the evidence

The certainty of the evidence was very low, especially due to problems of RoB, inconsistency, and imprecision (Supporting Information: Appendix 3 and Tables 1–4).

## 4 DISCUSSION

### 4.1 Main findings

The comorbidity of primary headaches with TMDs has been widely studied, considering that both entities act on each other as perpetuating and aggravating factors (Fernandes et al., 2013; Lim et al., 2010). Two of the included trials (Ekberg & Nilner, 2006; Goncalves et al., 2013) presented TMDs as comorbidity; however, in the study by Shankland (2002), patients with TMDs were excluded, despite the evidence of the overlap of symptoms between headaches and TMDs.

The present review analyzed two splint designs as conservative, nonpharmacological treatment of migraine and tension‐type headaches (Bruno & Krymchantowski, 2018; Ekberg & Nilner, 2006; Goncalves et al., 2013; Shankland, 2002). Based on the limited evidence, we cannot propose intraoral splints as the only therapeutic alternative for migraine and tension‐type headaches.

Although some of the studies (Goncalves et al., 2013) did observe a greater improvement in frequency, pain intensity, and disability when combining propranolol with intraoral splints relative to other groups (propranolol monotherapy, intraoral splints monotherapy, and placebo), the difference between groups on these outcomes did not reach statistical significance and clinical relevance. In addition, propranolol alone was not more effective in reducing any of the variables analyzed than the group that used stabilization splints. One possible explanation for these results could be that the prescribed dose of propranolol in this study is lower (90 mg daily) than the standard dose for the treatment of migraine (120–240 mg two to three times daily) (Ha & Gonzalez, 2019). Even so, these results suggest that females with migraine and TMDs could benefit from combination therapy since these headache outcomes improve when both conditions are treated regarding individualized treatment for each clinical entity (i.e. either migraine or TMD in isolation) (Goncalves et al., 2013). This idea has been confirmed by previous studies (Mitrirattanakul & Merrill, 2006) that examined the effects of headaches in patients with orofacial pain and found that primary headaches and musculoskeletal disorders could increase the pain present in the subjects, suggesting that these pathologies must be treated simultaneously to achieve satisfactory results.

It is important to mention that although no statistically significant differences were observed between intraoral and sham splints, a higher reduction of headache pain intensity was observed in patients receiving stabilization splints (Goncalves et al., 2013).

In none of the articles (Bruno & Krymchantowski, 2018; Shankland, 2002), adverse effects were reported related to the use of the NTI‐tss, such as occlusal changes due to over‐eruption posterior teeth, resulting in an open bite and/or the risk of choking with the appliances, being smaller in size compared with stabilization splints. Patients reported a good fit to this design of splints (Bruno & Krymchantowski, 2018), they did not experience sensitivity or mobility in the teeth. These findings are consistent with those observed by Jokstad et al. (2005) who did not detect occlusal alterations using these splints (Bruno & Krymchantowski, 2018; Shankland, 2002), probably due to their intermittent use during the day when the episodes occurred and during sleep. Also, no adverse events were reported when using the stabilization splints, unlike the pharmacological alternatives analyzed in the included studies (propranolol and amitriptyline), which reported drowsiness and light‐headedness (Bruno & Krymchantowski, 2018; Goncalves et al., 2013). Thus, intraoral splints would be a viable therapy that could be used for primary headaches, reducing perhaps the consumption of drugs with a lower risk of adverse events associated with them.

Regarding tension‐type headaches, the splints analyzed provided a positive effect compared to sham appliances (Ekberg & Nilner, 2006); the patients could benefit from these devices; however, more primary studies are needed with an adequate methodological design to obtain strong recommendations based on high‐quality evidence, the same was observed in previously published systematic reviews hence the need for more and better clinical research (Manrriquez et al., 2021; Stapelmann & Türp, 2008).

### 4.2 RoB

None of the studies (Bruno & Krymchantowski, 2018; Ekberg & Nilner, 2006; Goncalves et al., 2013; Shankland, 2002) presented a low RoB. Regarding the randomization process, they did not correctly describe it or did not directly report on it. Three studies did not report clear blinding for all study groups (Bruno & Krymchantowski, 2018; Ekberg & Nilner, 2006; Shankland, 2002); only one was considered a single‐blind study (Goncalves et al., 2013). Missing data could also affect the effect of the intervention in all four studies due to the high percentage of dropouts.

One study analyzed the primary outcomes at an intermediate time point before the completion of the study, which may contribute to an overestimation of the values obtained (Goncalves et al., 2013). The variables analyzed were frequency (primary variable), pain intensity, and disability (secondary variables), and the own patients evaluated them. The four included studies had severe methodological limitations (Bruno & Krymchantowski, 2018; Ekberg & Nilner, 2006; Goncalves et al., 2013; Shankland, 2002). There was heterogeneity between the studies, the study population varied between migraine, TTH, TMDs, or a combination of these entities with or without comorbidities associated. Two different diagnostic classifications were used; the ICHD‐2 (Olesen, 2004) in two studies (Bruno & Krymchantowski, 2018; Goncalves et al., 2013) and the HIS (Ravishankar, 2010) in another two (Ekberg & Nilner, 2006; Shankland, 2002), with only one study that included females exclusively (Goncalves et al., 2013). The follow‐up time was quite different between the studies and the measures for the analysis of the variables studied. For this reason, it was not possible to perform a meta‐analysis.

### 4.3 Certainty of the evidence

The certainty of the evidence was considered “very low” due to the high RoB, along with a severe imprecision of the estimates (small sample size) and heterogeneity of the included studies.

The number of patients included in each comparison analyzed was very small (<than the minimum of 300 subjects considered by the Cochrane Collaboration) (Schunemann, 2008) to determine no serious risk of precision. Two studies did not provide values so the effect could not be assessed (Ekberg & Nilner, 2006; Shankland, 2002), one of the studies presented wide intervals of confidence except for headache pain intensity (Goncalves et al., 2013); however, narrow intervals were identified for the frequency of headaches in the study by Bruno and Krymchantowski (2018). Although most of the domains of the GRADE approach were rated as very serious for most of the analyses, indirectness was considered appropriate since participants, interventions used, the comparisons made, and the variables analyzed provided direct evidence of the research question of this review.

Finally, it was not possible to assess publication bias due to the limited number of included studies.

### 4.4 Strengths and limitations

The main limitation of the present systematic review was the reduced number of RCTs and their high RoB. Furthermore, the studies included in this review presented great heterogeneity, especially in three factors: (1) diagnosis, (2) the design of the intraoral appliances, and (3) the outcomes' tool, which made it difficult to assess the quality of evidence of the review and did not allow a meta‐analysis. Another limitation is the short duration of the studies analyzed, except for one trial that analyzed the results at 12 months follow‐up (Ekberg & Nilner, 2006). The strengths of this review were a reliable methodology that followed strict standards, a comprehensive search strategy and up to date, the use of precise headache diagnoses, and analyzing relevant patient outcomes.

### 4.5 Future investigations

The quality of the included studies is very poor, so future RCTs conducted in this field need to control the biases highlighted in this review. In addition, larger sample sizes and longer follow‐up times are needed to determine the effectiveness of intraoral splints in reducing the frequency and intensity of primary headaches. In addition, it would be interesting to analyze other outcomes such as quality of life, satisfaction with treatment, and other psychosocial variables to see the impact of intraoral splints in other domains of patients' life. Furthermore, future studies should agree on diagnostic criteria to standardize the clinical picture and standardized outcome measures to determine the effectiveness and allow comparison between studies.

## 5 CONCLUSIONS

There is insufficient evidence to support the use of intraoral splints as a therapeutic alternative to pharmacology in treating migraine and/or tension‐type headache. The evidence supporting this claim is very poor due to the low quality and limited sample size of the studies included in this review. Thus, future well‐conducted randomized clinical trials are needed.

## AUTHOR CONTRIBUTIONS

**Olga Martínez‐Álvarez**: Contributed to conception, design, data acquisition, and interpretation, performed all statistical analyses, drafted, and critically revised the manuscript. **Eduardo Wojtovicz**: Contributed to data acquisition and interpretation and performed all statistical analyses. **Jose Luís De la Hoz**: Contributed to critically revised the manuscript. **Juan Mesa**: Contributed to critically revised the manuscript. **Susan Armijo‐Olivo**: Contributed to design, data acquisition, and interpretation and critically revised the manuscript.

## CONFLICT OF INTEREST STATEMENT

The authors declare no conflicts of interest.

## Supporting information

Supporting information.

## Data Availability

All data generated or analyzed during this study are included in this article and its supplementary information files (tables, figures, and appendices). The data published in the articles included in this systematic review have been used for analysis. Information that was not published in the original articles was not included.
