# Supplementary material for: Effectiveness of intraoral splints in the treatment of migraine and tension‐type headache: A systematic review
Source: Clin Exp Dent Res. 2023 Sep 7;9(6):1180–90. doi: 10.1002/cre2.779 (PMC10728529; doi:10.1002/cre2.779)
Supplement: Supplementary file 1 — Supporting information. [file CRE2-9-1180-s001.docx]

**Annexed 1. Electronic database search strategies.**

| **Electronic database** | **Search strategy** |
| --- | --- |
| ***Medline, PubMed, Embase, CINAHL PLUS with full text and Cochrane Library Trials* (searched up to March 16, 2021); re-run on June 12, 2022.** | *((splint OR occlusal splint OR oral splint OR oral appliance OR oral orthosis OR oral device) AND (migraine OR migraine disorders OR head pain OR primary headache OR headache OR headache disorders OR tension-type headache) AND (controlled clinical trial OR randomized study OR randomized controlled trial OR randomized trial OR randomized experiment OR clinical trial))* |
|  |  |
| **Scopus (searched up to March 16, 2021); re-run on Jun 12, 2022.** | *(( TITLE-ABS-KEY (controlled AND clinical W/3 trial)) OR (TITLE-ABS-KEY (randomized W/3 study )) OR ( TITLE-ABS-KEY ( randomized AND controlled W/3  trial )) OR ( TITLE-ABS-KEY (randomized  W/3  trial ))  OR  ( TITLE-ABS-KEY ( randomized W/3 experiment)) OR (TITLE-ABS-KEY ( clinical  W/3  trial ) ) )  AND  ( ( TITLE-ABS-KEY ( splint ) )  OR  ( TITLE-ABS-KEY ( occlusal  W/3  splint ) )  OR  ( TITLE-ABS-KEY ( oral  W/3  splint ) )  OR  ( TITLE-ABS-KEY ( oral  W/3  appliance ) )  OR  ( TITLE-ABS-KEY (oral W/3 orthosis)) OR (TITLE-ABS-KEY ( oral  W/3  device ) ) )  AND  ( ( TITLE-ABS-KEY ( migraine ) ) OR ( TITLE-ABS-KEY ( migraine  PRE/3  disorders ) )  OR  ( TITLE-ABS-KEY ( head  W/3  pain ) )  OR  ( TITLE-ABS-KEY ( primary  PRE/3  headache ) )  OR  ( TITLE-ABS-KEY ( headache ) )  OR  ( TITLE-ABS-KEY ( headache PRE/3 disorders )) OR ( TITLE-ABS-KEY ( tension-type  PRE/3  headache ) ) )* |
| ***Web of Science* (searched up to March 16 2021); re-run on June 12, 2022.** | *(((KP=controlled clinical trial OR KP= randomized study OR KP= randomized controlled trial OR KP=randomized trial OR KP=randomized experiment OR KP=clinical trial) OR (AK=controlled clinical trial OR AK= randomized study OR AK= randomized controlled trial OR AK=randomized trial OR AK=randomized experiment OR AK=clinical trial)) AND ((KP= migraine OR KP= migraine disorders OR KP= head pain OR KP= primary headache OR KP=headache OR KP=headache disorders OR KP=tension-type headache) OR (AK= migraine OR AK= migraine disorders OR AK= head pain OR AK= primary headache OR AK=headache OR AK=headache disorders OR AK=tension-type headache)) AND ((KP=splint OR KP=occlusal splint OR KP=oral splint OR KP= oral appliance OR KP=oral orthosis OR KP=oral device) OR (AK=splint OR AK=occlusal splint OR AK=oral splint OR AK= oral appliance OR AK= oral orthosis OR AK= oral device)))* |

| **Annexed 2. Reasons for exclusion** | |
| --- | --- |
| **First author, year. Title** | **Reason for exclusion** |
| **Blumenfeld and Boyd 2022. Adjunctive treatment of chronic migraine using an oral dental device: overview and results of a randomized placebo-controlled crossover study** | No classification of headache disorders or diagnosis by the neurologist |
| **de Tommaso *et al.,* 2005. Effects of amitriptyline and intra-oral device appliance on clinical and laser-evoked potentials features in chronic tension-type headache** | Inadequate design of intraoral splint |
| **de Tommaso *et al*., 2005. Effects of amitriptyline and intra-oral device appliance on clinical and laser-evoked potentials features in chronic tension-type headache** | Duplicate study |
| **Doepel *et al.,* 2011. Headache: short- and long-term effectiveness of a prefabricated appliance compared to a stabilization appliance** | Other headache disorder different from migraine and TTH |
| **Ekberg *et al*., 2002. Treatment outcome of headache after occlusal appliance therapy in a randomised controlled trial among patients with temporomandibular disorders of mainly arthrogenous origin** | Other headache disorder different from migraine and TTH |
| **Ekberg *et al.,* 2002. Treatment outcome of headache after occlusal appliance therapy in a randomized controlled trial among patients with temporomandibular disorders of mainly arthrogenous origin.** | Duplicate study |
| **Forssell *et al.* 1986. Effect of occlusal adjustment on mandibular dysfunction. A double-blind study** | No classification of headache disorders or diagnosis by the neurologist |
| **Haggiag *et al.,* 2020. A new biofeedback approach for the control of awake bruxism and chronic migraine headache: utilization of an awake posterior interocclusal device** | Inadequate design of intraoral splint |
| **Johansson *et al.,* 1991. Acupuncture in treatment of facial muscular pain** | Other headache disorder different from migraine and TTH |
| **Kostrzewa-Janicka *et al.,* 2013. Occlusal Stabilization Splint Therapy in Orofacial Pain and Tension-Type Headache** | Exclusion study design |
| **Lamey *et al.,* 1996. Migraine: the effect of acrylic appliance design on clinical response.** | Exclusion study design |
| **List *et al*., 1992. Acupuncture and occlusal splint therapy in the treatment of craniomandibular disorders .1. a comparative-study** | Other headache disorder different from migraine and TTH |
| **Michelotti *et al.,* 2012. Evaluation of the short-term effectiveness of education versus an occlusal splint for the treatment of myofascial pain of the jaw muscles** | Other headache disorder different from migraine and TTH |
| **Pelka *et al.,* 2001. Impulse magnetic-field therapy for migraine and other headaches: a double-blind, placebo-controlled study** | Oral splint does not appear as experimental group |
| **Porporatti *et al.,* 2015. Primary headaches interfere with the efficacy of temporomandibular disorders management** | Exclusion study design |
| **Quayle *et al.,* 1990. Soft occlusal splint therapy in the treatment of migraine and other headaches** | Use different classification to diagnosis |
| **Rampello *et al.,* 2013. A new aid in temporomandibular joint disorders' therapy: the universal neuromuscular immediate relaxing appliance.** | Other headache disorder different from migraine and TTH |
| **Rampello *et al.,* 2018. A novel universal device"LINGUAL RING Ri.P.A.Ra" for TMDs and cranio-cervico-mandibular pains: preliminary results of a randomized control clinical trial** | Other headache disorder different from migraine and TTH |
| **Rompre *et al.,* 2009. Reduction of morning headaches in subjects without sleep disordered breathing: an open study with a jaw retainer** | Other headache disorder different from migraine and TTH |
| **Saha *et al.,* 2019. Effects of occlusal splint therapy in patients with migraine or tension-type headache and comorbid temporomandibular disorder: a randomized controlled trial** | Use different classification to diagnosis |
| **Saha *et al.,* 2019. Effects of occlusal splint therapy in patients with migraine or tension-type headache and comorbid temporomandibular disorder A randomized controlled trial** | Duplicate study |
| **Sahebi *et al.,* 2018. The Effect of Flat Dual-Cure Stabilizer Occlusal Splint in Pain Relief of Individuals Suffering from Migraine Headaches** | Drug interaction |
| **Schokker *et al.,* 1990. The result of treatment of the masticatory system of chronic headache patients.** | Other headache disorder different from migraine and TTH |
| **ShanklandIi *et al.,* 2001. Migraine and Tension-Type Headache Reduction Through Pericranial Muscular Suppression: A Preliminary Report** | Exclusion study design |
| **ShanklandIi *et al.,* 2001. Migraine and Tension-Type Headache Reduction Through Pericranial Muscular Suppression: A Preliminary Report** | Duplicate study |
| **Shankland *et al.,* 2001. Nociceptive trigeminal inhibition--tension suppression system: a method of preventing migraine and tension headaches** | Corrected and republished study |
| **Shankland *et al.,* 2002. Nociceptive trigeminal inhibition--tension suppression system: a method of preventing migraine and tension headaches** | Duplicate study |
| **Vallon *et al.,* 1991. Short-term effect of occlusal adjustment on craniomandibular disorders including headaches.** | Other headache disorder different from migraine and TTH |
| **Wassell *et al.,* 2004. Treatment of temporomandibular disorders by stabilising splints in general dental practice: results after initial treatment** | Other headache disorder different from migraine and TTH |
| **Wenneberg *et al.,* 1988. Occlusal equilibration and other stomatognathic treatment in patients with mandibular dysfunction and headache** | No classification of headache disorders or diagnosis by the neurologist |
| *Annexed 2. Reasons for exclusion of the selected articles for full-text reading. Abbreviations: TTH, tension-type headache.* | |

**Annexed 3. Quality evidence (GRADE)**

**Annexed 3. Table 1. GRADE**

**Question:** Oral splints compared to sham splints for migraine treatment

| **Certainty assessment** | | | | | | | **№ of patients** | | **Effect** | | **Certainty** | **Importance** |
| --- | --- | --- | --- | --- | --- | --- | --- | --- | --- | --- | --- | --- |
| **№ of studies** | **Study design** | **Risk of bias** | **Inconsistency** | **Indirectness** | **Imprecision** | **Other considerations** | **Oral splints** | **Sham splints** | **Relative (95% CI)** | **Absolute (95% CI)** |  |  |
| **Frequency headache (follow-up: 8 weeks to 3 months; assessed with: Daily calendars)** | | | | | | | | | | | | |
| 2 studies  Gonçalves *et al.,* 2013  Bruno & Krymchantowski, 2018 | randomised trials | serious^a^  serious^e^ | not serious^b^ | not serious^c^ | serious^d^ | none | 48 | 44 | - | SMD: 0.27 [-0.33, 0.86]  SMD: 0.00 [-0.57, 0.57] | ⨁◯◯◯ Very low | CRITICAL |
| **Intensity headache (follow-up: 8 weeks to 3 months; assessed with: Visual analog scale (VAS); Scale from: 0 to 100)** | | | | | | | | | | | | |
| 1 study  Gonçalves *et al.,* 2013 | randomised trial | serious^a^ | NA^f^ | not serious^c^ | serious^d^ | none | 23 | 21 | - | SMD: -0.49 [-1.09, 0.11] | ⨁◯◯◯ Very low | CRITICAL |
| **Disability headache (follow-up: mean 3 months; assessed with: MIDAS scale)** | | | | | | | | | | | | |
| 1 study  Gonçalves *et al.,* 2013 | randomised trial | serious^a^ | NA^f^ | not serious^c^ | serious^d^ | none | 23 | 21 | - | SMD: 003 [-0.57, 0.62] | ⨁◯◯◯ Very low | CRITICAL |

***CI:*** *confidence interval;* ***NA:*** *not applicable*

***Explanations***

*a. Loss of data could affect the effect of the intervention. The variables were analyzed at a follow-up time prior to the end of the study, which could contribute to an overestimation of the values ​​obtained. The assessment was categorized as high risk of bias.*

*b. Differences in direction but minimal heterogeneity*

*c. The comparison coincides with the PICO format of this systematic review.*

*d. The confidence intervals are wide and the sample size is small.*

*e. Although both groups were blinded and highly motivated, the risk of bias was judged high for several reasons: dropouts were not included in the statistical analysis, method of randomization and outcome measures were not adequately described.*

*f. Heterogeneity is not evaluable as it is a single study. It can also be considered as a lack of evidence.*

**Annexed 3. Table 2. GRADE**

**Question:** Oral splints compared to other interventions for migraine treatment

| **Certainty assessment** | | | | | | | **№ of patients** | | **Effect** | | **Certainty** | **Importance** |
| --- | --- | --- | --- | --- | --- | --- | --- | --- | --- | --- | --- | --- |
| **№ of studies** | **Study design** | **Risk of bias** | **Inconsistency** | **Indirectness** | **Imprecision** | **Other considerations** | **Oral splints** | **Other intervention** | **Relative (95% CI)** | **Absolute (95% CI)** |  |  |
| **Frequency headache (follow-up: mean 3 months; assessed with: Daily calendars)** | | | | | | | | | | | | |
| 2 studies  Gonçalves *et al.,* 2013  Bruno & Krymchantowski, 2018 | randomised trials | serious^a^  serious ^e^ | serious^b^ | not serious^c^ | serious^d^ | none | 48 | 51 | - | SMD: 0.08 [-0.50, 0.66]  SMD: 0.73 [0.17, 1.29] | ⨁◯◯◯ Very low | CRITICAL |
| **Intensity headache (follow-up: mean 3 months; assessed with: Visual analog scale (VAS); Scale from: 0 to 100)** | | | | | | | | | | | | |
| 1 study  Gonçalves *et al.,* 2013 | randomised trial | serious^a^ | NA^f^ | not serious^c^ | serious^d^ | none | 23 | 23 | - | SMD: -0.14 [-0.72, 0.44] | ⨁◯◯◯ Very low | CRITICAL |
| **Disability headache (assessed with: MIDAS scale)** | | | | | | | | | | | | |
| 1 study  Gonçalves *et al.,* 2013 | randomised trial | serious^a^ | NA^f^ | not serious^c^ | serious^d^ | none | 23 | 23 | - | SMD: -0.27 [-0.85, 031] | ⨁◯◯◯ Very low | CRITICAL |

***CI:*** *confidence interval;* ***NA:*** *not applicable*

***Explanations***

*a. Loss of data could affect the effect of the intervention. The variables were analyzed in a follow-up time prior to the end of the study, which could contribute to an overestimation of the values ​​obtained. The assessment was rated as high risk of bias.*

*b. The magnitude of the variability of the results is similar, the two studies suggest a benefit. It presents inconsistency.*

*c. The comparison coincides with the PICO format of this systematic review*

*d. Wide confidence intervals and small sample size. All this indicates little precision.*

*e. It was judged to be at high risk of bias for several reasons: the amitriptyline group was not blinded, dropouts were not included in the statistical analysis, the method of randomization, and the outcome measures were poorly described.*

*f. Heterogeneity is not assessable as it is a single study. It can also be considered a as lack of evidence.*

**Annexed 3. Table 3. GRADE**

**Question:** Oral splints compared to sham splints for tension-type headache treatment

| **Certainty assessment** | | | | | | | **№ of patients** | | **Effect** | | **Certainty** | **Importance** |
| --- | --- | --- | --- | --- | --- | --- | --- | --- | --- | --- | --- | --- |
| **№ of studies** | **Study design** | **Risk of bias** | **Inconsistency** | **Indirectness** | **Imprecision** | **Other considerations** | **Oral splints** | **Sham splints** | **Relative (95% CI)** | **Absolute (95% CI)** |  |  |
| **Frequency headache (follow-up: mean 2 months; assessed with: Daily calendars)** | | | | | | | | | | | | |
| 1 study  Ekberg & Nilner, 2006 | randomised trial | serious^a^ | NA^b^ | not serious^c^ | very serious^d^ | none | 30 | 10 | - | Not Available | ⨁◯◯◯ Very low | CRITICAL |

***CI:*** *confidence interval;* ***NA:*** *not applicable*

***Explanations***

*a. The high risk of bias assessment. Possible loss of blinding of patients once allocation was known due to difficulty in maintaining blinding when comparing two oral devices. A significant number of dropouts were in the control group.*

*b. Heterogeneity is not assessable as it is a single study. It can also be considered as a lack of evidence.*

*c. Matches the PICO format of the research question*

*d. It does not report the effect size or confidence intervals.*

**Annexed 3. Table 4. GRADE**

**Question:** Oral splints compared to sham splints for tension-type headache and migraine treatment

| **Certainty assessment** | | | | | | | **№ of patients** | | **Effect** | | **Certainty** | **Importance** |
| --- | --- | --- | --- | --- | --- | --- | --- | --- | --- | --- | --- | --- |
| **№ of studies** | **Study design** | **Risk of bias** | **Inconsistency** | **Indirectness** | **Imprecision** | **Other considerations** | **Oral splints** | **Sham splints** | **Relative (95% CI)** | **Absolute (95% CI)** |  |  |
| **Frequency headache (follow-up: mean 2 months; assessed with: Daily calendars)** | | | | | | | | | | | | |
| 1 study  Shankland, 2002 | randomised trial | very serious^a^ | NA^b^ | not serious^c^ | very serious^d^ | none | 51 | 43 | - | **Not available** | ⨁◯◯◯ Very low | CRITICAL |
| **Intensity headache (assessed with: Visual analog scale (VAS); Scale from: 0 to 100)** | | | | | | | | | | | | |
| 1 study  Shankland, 2002 | randomised trial | very serious^a^ | NA^b^ | not serious^c^ | very serious^d^ | none | 51 | 43 | - | **Not available** | ⨁◯◯◯ Very low | CRITICAL |

***CI:*** *confidence interval;* ***NA:*** *not applicable*

***Explanations***

*a. This study presents low methodological quality with a high risk of bias. It does not describe and the process of randomization and blinding of patients is not clear, they do not report dropouts and insufficient reporting of results.*

*b. Heterogeneity is not assessable as it is a single study. It can also be considered as a lack of evidence.*

*c. Matches the PICO format of the research question.*

*d. It does not report the effect size or confidence intervals. It also presents a small non-individualized sample size of the migraine and TTH entity.*
